# Genotype-Dependent Variations in Oxidative Stress Markers and Bioactive Proteins in Hereford Bulls: Associations with *DGAT1*, *LEP*, and *SCD1* Genes

**DOI:** 10.3390/biom14101309

**Published:** 2024-10-16

**Authors:** Piotr Kostusiak, Emilia Bagnicka, Beata Żelazowska, Magdalena Zalewska, Tomasz Sakowski, Jan Slósarz, Marcin Gołębiewski, Kamila Puppel

**Affiliations:** 1 Institute of Animal Science, Warsaw University of Life Sciences, Ciszewskiego 8, 02-786 Warsaw, Poland; piotr_kostusiak@sggw.edu.pl (P.K.); jan_slosarz@sggw.edu.pl (J.S.); marcin_golebiewski@sggw.edu.pl (M.G.); 2 Institute of Genetics and Animal Biotechnology, Polish Academy of Science, Jastrzębiec, Postępu 36A, 05-552 Magdalenka, Poland; e.bagnicka@igbzpan.pl (E.B.); b.zelazowska@igbzpan.pl (B.Ż.); t.sakowski@igbzpan.pl (T.S.); 3 Department of Bacterial Physiology, Institute of Microbiology, Faculty of Biology, University of Warsaw, 02-096 Warsaw, Poland

**Keywords:** genetic variations, *DGAT1*, *LEP*, *SCD1*, oxidative stress, beef

## Abstract

The objective of this study is to assess the influence of genetic polymorphisms in *DGAT1*, *LEP*, and *SCD1* on the oxidative stress biomarkers and bioactive protein levels in Hereford bulls. A total of sixty-eight bulls were analyzed at 22 months of age to assess growth metrics and carcass quality, with a focus on polymorphisms in these genes. The key markers of oxidative stress, including malondialdehyde (MDA), and the activities of antioxidant enzymes such as glutathione reductase (GluRed), glutathione peroxidase (GPx), and superoxide dismutase (SOD) were measured, alongside bioactive compounds like taurine, carnosine, and anserine. The results show that the TT genotype of *DGAT1* is linked to significantly higher MDA levels, reflecting increased lipid peroxidation, but is also associated with higher GluRed and GPx activities and elevated levels of taurine, carnosine, and anserine, suggesting an adaptive response to oxidative stress. The *LEP* gene analysis revealed that the CC genotype had the highest MDA levels but also exhibited increased GPx and SOD activities, with the CT genotype showing the highest SOD activity and the TT genotype the highest total antioxidant status (TAS). The *SCD1* AA genotype displayed the highest activities of GluRed, GPx, and SOD, indicating a more effective antioxidant defence, while the VA genotype had the highest MDA levels and the VV genotype showed lower MDA levels, suggesting protective effects against oxidative damage. These findings highlight genotype specific variations in the oxidative stress markers and bioactive compound levels, providing insights into the genetic regulation of oxidative stress and antioxidant defences, which could inform breeding strategies for improving oxidative stress resistance in livestock and managing related conditions.

## 1. Introduction

Several point mutations in genes have been identified, with significant effects on cattle phenotypes. Among these, leptin, a polypeptide hormone predominantly secreted by adipocytes, plays a crucial role in energy homeostasis by modulating appetite and lipid metabolism [1]. The leptin gene in cattle is located on chromosome 4, where several polymorphisms associated with performance traits have been identified [2]. Studies indicate that animals with the TT genotype exhibit superior sensory preference scores compared to those with CC or CT genotypes. Additionally, SNP UASMS2 shows a significant correlation with fat thickness, where CC-genotype animals display reduced fat surrounding the loin compared to the CT or TT genotypes [3]. Diacylglycerol O-acyltransferase (*DGAT1*) is a key microsomal enzyme catalyzing the final step in triglyceride synthesis, converting diacylglycerol and fatty acids into triglycerides. The K232A SNP involves a dinucleotide substitution (AA/GC), resulting in the amino acid substitution of lysine for alanine. Investigations have demonstrated that SNP c.572 A > G is linked with variations in fat thickness, MBS (meat quality score), fat colour, and shear force [4,5]. Specifically, animals with the BB genotype exhibit lower MBS values, reduced fat colour, and lower shear force compared to those with the AA genotype. Stearoyl-CoA desaturase (*SCD1*) catalyzes the conversion of saturated fatty acids (SFA) to unsaturated fatty acids (UFA) in adipocytes by introducing double bonds. *SCD* activity is regulated by key lipogenic enzymes involved in fatty acid synthesis pathways, with leptin and insulin also modulating its activity. In the gene encoding *SCD1* on chromosome 26, three SNPs in the 3′UTR and three SNPs in exon 5 have been identified. Significant associations have been observed between the g.10329 C > T polymorphism and the desaturation index and fatty acid composition [6]. Additionally, polymorphisms in *SCD1* are significantly correlated with meat sensory traits, such as colour, tenderness, and taste perception. The *SCD1* gene is a critical determinant of meat sweetness, with heterozygous genotypes showing higher sensory ratings compared to homozygous VV genotypes [7].

Enzymes, substrates, and coenzymes are compartmentalized within specialized subcellular structures, allowing for precise and selective biochemical activity [8]. This compartmentalization enables the isolation and optimization of opposing biochemical processes, such as the biosynthesis and degradation of metabolites [9,10]. The activity of enzymes in blood serum is influenced by various factors, including pathological conditions, the extent of tissue damage, and the rate of catabolism and clearance from the circulatory system. Enzymes are distributed across distinct subcellular compartments, including in the cytoplasm (e.g., aldolase, phosphohexose isomerase, lactate dehydrogenase, alanine aminotransferase, sorbitol dehydrogenase), mitochondria (e.g., Krebs cycle enzymes, oxidases, glutamate dehydrogenase, aspartate aminotransferase), endoplasmic reticulum (e.g., esterases, reductases, acetylases, gamma-glutamyltransferase), ribosomes (e.g., protein biosynthesis enzymes, ceruloplasmin, cholinesterase), and lysosomes (e.g., proteases, phosphatases, collagenases). In response to oxidative stress, cattle utilize two primary defence mechanisms, namely enzymatic and non-enzymatic [11]. Enzymatic antioxidants, which facilitate redox reactions, are categorized into the following four major groups: oxidases, dehydrogenases, peroxidases, and oxygenases [12]. Key examples include superoxide dismutase (SOD), which catalyzes the disproportionation of superoxide anions [13]; glutathione peroxidase (GPx), which decomposes peroxides and mitigates oxidative damage [14]; and glutathione reductase (GluRed), which regenerates reduced glutathione [15]. Non-enzymatic defence mechanisms involve proteins with intrinsic antioxidant properties, which contribute to the cellular protection against oxidative damage [16].

Carnosine, a dipeptide composed of β-alanine and histidine, fulfils several critical biological functions. It exhibits chelating activity by forming stable complexes with heavy metal cations, which mitigates their toxic effects [17]. The antioxidant properties of carnosine are attributed to its ability to neutralize ROS [18], including hydroxyl radicals and peroxide radicals, thereby mitigating oxidative stress [19]. Furthermore, carnosine has antiglycative properties, as it inhibits glycation reactions in which low molecular weight aldehydes, such as methylglyoxal, cause damage to protein structures [20,21]. Anserine, a methylated derivative of carnosine consisting of β-alanine and L-(N-methyl)histidine, also functions as an antioxidant. It provides cellular protection against oxidative damage and contributes to the maintenance of homeostasis in muscle and brain tissues [22].

Oxidative stress in beef cattle arises from an imbalance between reactive oxidative species and the capacity for detoxification and repair mechanisms [9]. This imbalance correlates with diminished immune function and increased disease vulnerability [12]. Consequently, elevating antioxidant defences is essential for enhancing animal health and improving the oxidative stability of meat [23].

The aim of this study is to assess the influence of genetic polymorphisms in *DGAT1*, *LEP*, and *SCD1* on oxidative stress biomarkers and bioactive protein levels in Hereford bulls. The research aims to quantify how variations in these genes correlate with changes in oxidative stress markers such as malondialdehyde, glutathione reductase, glutathione peroxidase, superoxide dismutase, and total antioxidant status, as well as with concentrations of bioactive compounds including taurine, anserine, carnosine, and coenzyme Q10. This investigation seeks to identify the genetic factors that modulate oxidative stress responses and bioactive protein profiles, thereby contributing to the understanding of genetic influences on meat quality and overall health in Hereford cattle.

## 2. Materials and Methods

### 2.1. Animals

The study involved 65 Hereford bulls, aiming to evaluate growth performance and muscle characteristics in relation to genetic variations (Table S1). The bulls were slaughtered at 22 months of age, selected to ensure maturity for assessing growth metrics and carcass quality. Prior to slaughter, the standardized live weight was recorded with an average of 669 kg, and carcass weight was measured post-slaughter, averaging 390 kg. After slaughter, the carcasses were cooled for 24 h at temperatures between 2 and 4 °C. Following the cooling period, a muscle sample weighing 300 g was collected from the semimembranosus muscle, sampled parallel to the muscle axis to ensure consistency in the analysis of muscle composition.

All bulls had unlimited access to pasture for 1 year of their life. After the grazing phase, the animals had transition and finishing rations (Table S2). To get the animals used to a changed diet, two step-up diets were introduced. The average standardized daily gain was 1.04 kg/day, and the daily carcass gain was 0.66 kg/day.

Additionally, EUROP trade grades and fat classes were estimated by graders at the slaughterhouse. The carcasses were classified, with fat classes ranging from 1 to 2+, and trade classes ranging from U to R. This classification provided insights into the quality and fat content of the carcasses, contributing to a comprehensive evaluation of growth performance and muscle characteristics in relation to genetic variations.

### 2.2. Chemical Analyses

Beef samples were initially chopped and then processed in a blender to achieve a homogeneous mixture. This homogenized sample was analyzed using a near-infrared spectrophotometer. The basic chemical composition of the meat was determined utilizing a Food Scan™ analyser (Foss Electric, Hillerød, Denmark).

The quantification of anserine, carnosine, taurine, and coenzyme Q10 was performed using an RP-HPLC Agilent 1100 system. Chromatographic separations were executed at ambient temperature employing a solvent gradient on a Jupiter C18 300A column (Phenomenex, Torrance, CA, USA), following the procedure outlined by Łukasiewicz et al. [24]. The mobile phases comprised Solvent A, which included acetonitrile (Merck, Darmstadt, Germany), water (Sigma-Aldrich), and trifluoroacetic acid (Sigma-Aldrich, St. Louis, MO, USA) in a volumetric ratio of 30:970:1 (*v*/*v*/*v*), and Solvent B, which consisted of acetonitrile, water, and trifluoroacetic acid in a ratio of 970:30:1 (*v*/*v*/*v*). The flow rate was set at 1.4 mL/min, with detection occurring at a wavelength of 214 nm. A 25 µL aliquot of the final solution was injected for analysis. All samples were analyzed in duplicate to ensure reproducibility. Peak identification and quantification were verified through comparison with standards (Sigma-Aldrich, St. Louis, MO, USA).

The levels of malondialdehyde (MDA) in blood plasma were determined using a NanoQuant Infinite M200 Pro analyser (Tecan Austria GmbH, Grödig, Austria) at a wavelength of 532 nm. In this process, 250 µL of blood plasma was mixed with 25 µL of 0.2% 2,6-bis(1,1-dimethyl-ethyl)-4-methylphenol (Sigma-Aldrich, Warsaw, Poland) and 1 mL of 5% trichloroacetic acid (Sigma-Aldrich, Warsaw, Poland). The mixture was centrifuged at 14,000× *g* for 10 min. Subsequently, 750 µL of the clear supernatant was transferred to a glass tube, and 500 µL of 0.6% thiobarbituric acid (Sigma-Aldrich, Warsaw, Poland) was added. The solution was mixed and incubated for 45 min in a 90 °C water bath. After incubation, the mixture was cooled on ice and centrifuged at 4000× *g* for 5 min. Finally, 200 µL of the clear supernatant was transferred to a microplate for measurement.

The glutathione reductase (GluRed), glutathione peroxidase (GPx), superoxide dismutase (SOD), and total antioxidant status (TAS) in blood plasma were assessed using a NanoQuant Infinite M200 Pro analyser (Tecan Austria GmbH, Grödig, Austria) with dedicated ELISA kits from Randox Laboratories (Crumlin, UK). The specific kits used were as follows: glutathione reductase (Cat no GR2608), Ransel (glutathione peroxidase, Cat no SC692), Ransod (superoxide dismutase, Cat no SD126), and Total Antioxidant Status (Cat no NX2331).

### 2.3. DNA Sampling and Analysis

Blood samples were collected in tubes containing anticoagulants for genetic analysis. Total DNA was isolated from whole blood using the DNeasy Blood & Tissue kit (Qiagen, Hilden, Germany), following the manufacturer’s protocol. The quantity of the extracted DNA was measured with an Invitrogen Qubit 4.0 Fluorometer (Thermo Fisher Scientific, Waltham, MA, USA) using a dsDNA high-sensitivity assay kit, while the quality was assessed with a NanoDrop 2000 spectrophotometer (Thermo Fisher Scientific, Waltham, MA, USA). DNA samples were isolated in triplicate and then pooled for each animal to ensure representative and reliable genetic analysis. DNA isolation and genotyping were conducted at the Institute of Genetics and Animal Biotechnology PAS, Jastrzębiec, Poland, using the Restriction Fragment Length Polymorphism (RFLP) method. The amplifications of the *SCD1* gene were performed using a forward primer 5′-ATG TAT GGA TAC CGC CCT TAT-3′ and a reverse primer 5′-TTC TGG CAC GTA ACC TAT ACC CT-3′. The restriction enzyme Fnu4HI, that cuts at the 5′...GC|NGC...3′ site, has been used to digest the 145 bp amplicon. A T > C polymorphism genotypes resulted in different fragment patterns, as follows: the genotype AA in fragments of 29, 48 and 68 bp, the genotype VA in 29, 48, 68 and 116 bp, and the genotype VV showed fragments of 29 and 116 bp [25]. The *LEP* gene used the primers 5′-ATG CGT GTG GAC CCC TGT ATC-3′ (forward) and 5′-TGG TGT CAT CCT GGA CCT CC-3′ (reverse) to amplify the 94 bp product. The enzyme BspEI was used to digest the amplicon, cutting at the 5′...T|CCGGA...3′ site. The T > C polymorphism resulted in different fragment patterns for each genotype, as follows: the CC genotype fragments were 75 bp, the CT genotype fragments were 75 and 94 bp, and the TT genotype fragments were 94 bp [26]. The amplification of the *DGAT1* gene was performed using the forward primer 5′-GCA CCA TCC TCT TCC TCA AG-3′ and the reverse primer 5′-GGA AGC GCT TTC GGA TG-3′. The restriction enzyme CfrI, which cuts the sequence 5′...Y|GGCCR...3′, was used to digest an amplicon of 411 bp. T > C substitution resulted in a polymorphism in which the CC genotype remained undigested at 411 bp, the CT genotype resulted in fragments of 203 and 411 bp, and the TT genotype yielded fragments of 203 bp [27]. The genes selected for analysis were Stearoyl-CoA desaturase (*SCD1*), leptin (*LEP*), and diacylglycerol O-acyltransferase (*DGAT1*), as these genes are associated with key qualitative traits in beef. Polymerase Chain Reaction (PCR) was performed using AmpliTaq Gold DNA Master Mix (Thermo Fisher Scientific, Waltham, MA, USA). PCR conditions were specifically optimized for each reaction in accordance with the manufacturer’s protocol for the polymerase.

### 2.4. Statistical Analysis

Variance analysis (ANOVA) was performed to evaluate the impact of genetic polymorphisms on the biomarkers and protein levels. This analysis allowed for the comparison of means among different genotypes and the identification of statistically significant differences. To evaluate the impact of genetic polymorphisms on oxidative stress biomarkers and bioactive protein levels, we conducted an analysis of variance (ANOVA). Prior to performing the ANOVA, the normality of the data distribution was assessed using the Shapiro–Wilk test for all variables, including oxidative stress markers such as malondialdehyde (MDA), glutathione reductase (GluRed), glutathione peroxidase (GPx), superoxide dismutase (SOD), and total antioxidant status (TAS), as well as for bioactive compounds such as taurine, anserine, and carnosine.

The results of the Shapiro–Wilk test confirmed that the data were normally distributed (*p* > 0.05 for all variables), allowing us to proceed with parametric tests. If any of the variables had deviated from normality, we would have used non-parametric tests. However, as all datasets met the normality criteria, ANOVA was deemed appropriate.

In addition to ANOVA, Pearson’s correlation coefficients were calculated to explore the linear relationships between genetic polymorphisms (*DGAT1*, *LEP*, and *SCD1*) and oxidative stress biomarkers. Spearman’s correlation coefficients were used to examine potential non-linear associations between genetic variants and bioactive protein levels. Only statistically significant interactions with *p*-values of ≤0.01 or ≤0.05 were considered for detailed interpretation.

All statistical analyses were conducted using IBM SPSS Statistics version 23 software to ensure the accuracy and reliability of the results.

## 3. Results

**Figure 1:**
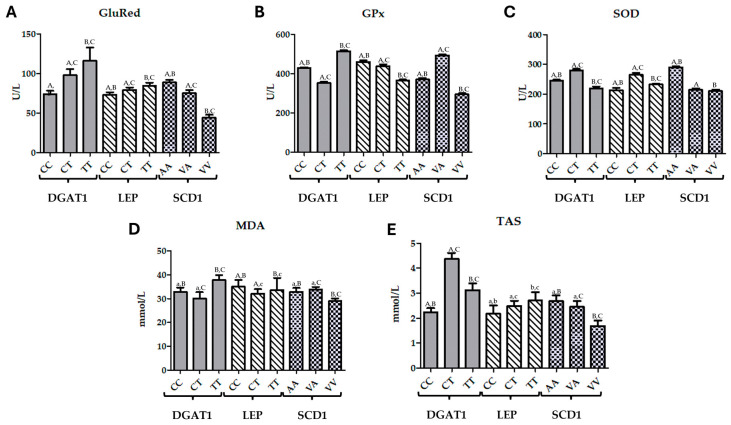
The effect of DGAT1 (CC, CT, TT), LEP (CC, CT, TT), SCD1 (AA, VA, VV) genetic variants on the levels of (**A**) glutathione reductase (GluRed), (**B**) glutathione peroxidase (GPx), (**C**) superoxide dismutase (SOD), (**D**) malondialdehyde (MDA), and (**E**) total antioxidant status (TAS) was assessed. Data are presented as figures of Last Square Means ± SEM; values with the same letters in one gene group differ significantly: upper case indicates *p* ≤ 0.01; lower case indicates *p* ≤ 0.05.

The studies conducted on Hereford bulls revealed genotype-dependent variations in oxidative stress biomarkers across the *DGAT1*, *LEP*, and *SCD1* genes (Figure 1).

For *DGAT1*, the TT genotype showed the highest MDA levels, with an increase of 15% compared to the CC genotype and 26% compared to the CT genotype. GluRed activity was also highest in the TT genotype, with a 57% increase over the CC genotype and 19% over the CT genotype. GPx activity in the TT genotype was the most elevated, with a 20% increase compared to the CC genotype and 46% compared to the CT genotype. Interestingly, SOD activity peaked in the CT genotype, being 14% higher than in the CC genotype and 28% higher than in the TT genotype. TAS was most elevated in the CT genotype, with a 95% increase compared to the CC genotype and 40% compared to the TT genotype.

For the *LEP* gene, the CC genotype had the highest MDA levels, showing a 9% increase over the TT genotype and 10% over the CT genotype. GluRed activity peaked in the TT genotype, with a 16% increase over the CC genotype and 7% over the CT genotype. GPx activity was highest in the CC genotype, with a 5% increase over the CT genotype and a 26% increase over the TT genotype. SOD activity was greatest in the CT genotype, showing a 24% increase over the CC genotype and 14% over the TT genotype. TAS was highest in the TT genotype, with a 24% increase over the CC genotype and 9% over the CT genotype (Figure 1).

For the *SCD1* gene, the VA genotype had the highest MDA levels, with a 3% increase compared to the AA genotype, while the VV genotype had the lowest levels, with a decrease of 14% compared to AA and 17% compared to VA. GluRed activity was highest in the AA genotype, showing an 18% increase over the VA genotype and a significant 50% increase over the VV genotype. GPx activity peaked in the VA genotype, with a 33% increase compared to the AA genotype and 68% over the VV genotype. SOD activity was highest in the AA genotype, being 35% higher than the VA genotype and 38% higher than the VV genotype. TAS was also highest in the AA genotype, with a 9% increase over the VA genotype and a 59% increase compared to the VV genotype (Figure 1).

In summary, the data reveal significant genotype-dependent variations in oxidative stress biomarkers across the *DGAT1*, *LEP*, and *SCD1* genes. The TT genotype of *DGAT1* is associated with higher oxidative stress markers, as indicated by elevated MDA and GPx levels. For *LEP*, the CC genotype is correlated with higher oxidative stress, while for *SCD1*, the AA genotype is linked to increased antioxidant enzyme activities and higher TAS, suggesting a stronger antioxidant defence. These findings underscore the role of genetic polymorphisms in modulating oxidative stress and antioxidant responses.

The correlation analysis of oxidative stress markers with *DGAT1*, *LEP*, and *SCD1* gene variations reveals specific significant relationships. For the *DGAT1* gene, a significant positive correlation was observed with GluRed activity, with a correlation coefficient of 0.369 (*p* < 0.01). This suggests that variations in *DGAT1* are associated with increased GluRed activity, reflecting enhanced antioxidant defence. Additionally, *DGAT1* showed a significant positive correlation with TAS, with a correlation coefficient of 0.309 (*p* < 0.05), indicating that *DGAT1* variations are linked to higher overall antioxidant capacity (Figure 1).

In contrast, the *LEP* gene did not show significant correlations with MDA, GluRed, GPx, and SOD. However, *LEP* exhibited significant negative correlations with taurine (−0.578, *p* < 0.01) and anserine (−0.514, *p* < 0.01), indicating substantial inverse relationships with these bioactive compounds (Table 1).

**Figure 2:**
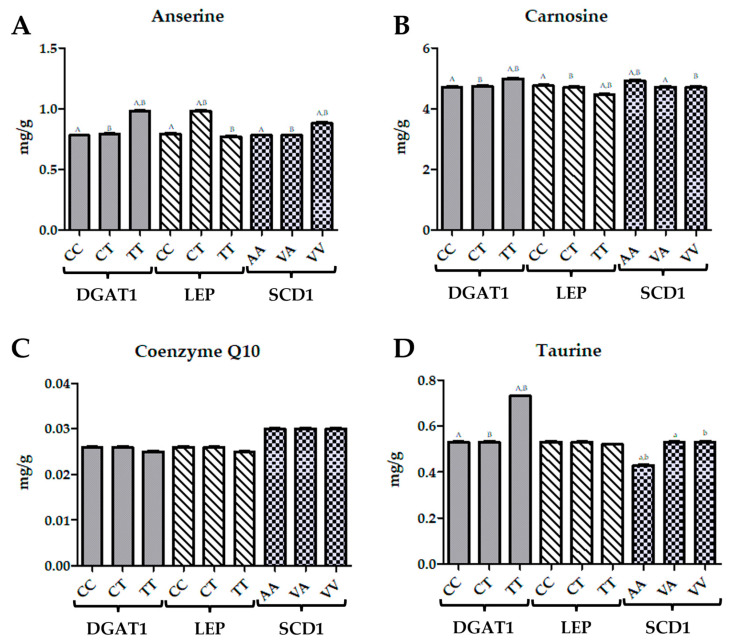
The effect of the genetic variants of DGAT1 (CC, CT, TT), LEP (CC, CT, TT), SCD1 (AA, VA, VV) on levels of (**A**) anserine, (**B**) carnosine, (**C**) coenzyme Q10, (**D**) taurine. Data presented as figures of Last Square Means ± SEM; values with the same letters in one gene group differ significantly: upper case indicates *p* ≤ 0.01; lower case indicates *p* ≤ 0.05.

For the *SCD1* gene, significant negative correlations were found with GluRed and SOD, with correlation coefficients of −0.318 (*p* < 0.05) and −0.368 (*p* < 0.01), respectively. These correlations indicate that variations in *SCD1* are associated with decreased antioxidant enzyme activities (Figure 2). Additionally, *SCD1* had a significant negative correlation with total antioxidant status (TAS) at −0.275 (*p* < 0.05), suggesting a moderate inverse relationship with overall antioxidant capacity (Table 1).

For the *DGAT1* gene, taurine levels were highest in the TT genotype, showing a 38% increase compared to the CC and CT genotypes. Q10 levels remained consistent across all genotypes, with no significant differences (Figure 2). Carnosine concentrations were highest in the TT genotype, representing an 8% increase compared to the CC genotype and a 12% increase compared to the CT genotype. Anserine levels were also highest in the TT genotype, showing a 26% increase compared to the CC genotype and a 15% increase compared to the CT genotype. In the case of the *LEP* gene, taurine levels were stable across the CC and CT genotypes, but were slightly lower in the TT genotype by 2%. Q10 concentrations were uniform across all genotypes. Carnosine levels were highest in the CC genotype, showing a 6% increase compared to the TT genotype. Anserine concentrations were highest in the TT genotype, with a 24% increase compared to the CC genotype and a 27% increase compared to the CT genotype. For the *SCD1* gene, taurine levels were lowest in the AA genotype, with a 19% decrease compared to the CC and CT genotypes and a 19% decrease compared to the VA and VV genotypes. Q10 levels were highest in the AA, VA, and VV genotypes, with no significant differences among them. Carnosine concentrations were highest in the AA genotype, with a 4% increase compared to the VA genotype and a 10% increase compared to the VV genotype. Anserine levels were highest in the VV genotype, showing a 13% increase compared to the AA genotype and a 13% increase compared to the VA genotype (Figure 2).

The correlation analysis conducted for taurine, Q10, carnosine, and anserine concentrations with respect to the *DGAT1*, *LEP*, and *SCD1* genes provides insights into the relationships between these biomarkers and genetic variations (Table 2). For *DGAT1*, the correlations with taurine, Q10, carnosine, and anserine were negative but not statistically significant, with values of −0.055, −0.078, −0.051, and −0.062, respectively. This indicates a lack of a strong linear relationship between *DGAT1* gene variations and these biomarkers. In contrast, for the *LEP* gene, significant negative correlations were observed. Taurine showed a strong negative correlation of −0.578, which is significant at the 0.01 level, suggesting a notable inverse relationship between *LEP* and taurine concentrations. Similarly, Q10 had a significant negative correlation of −0.411 at the 0.05 level, indicating a moderate inverse relationship. Carnosine and anserine also exhibited negative correlations with *LEP*, with values of −0.278 and −0.514, respectively. The correlation for carnosine is significant at the 0.05 level, while anserine’s correlation is significant at the 0.01 level, both indicating substantial inverse relationships. For the *SCD1* gene, the correlations with taurine, Q10, carnosine, and anserine were all negative but not statistically significant, with values of −0.024, −0.037, −0.025, and −0.014, respectively. These correlations suggest a weak inverse relationship between *SCD1* gene variations and the biomarker concentrations (Table 2).

## 4. Discussion

*DGAT1* Gene

The TT genotype of the *DGAT1* gene is associated with significantly elevated malondialdehyde MDA levels, showing a 15% increase compared to the CC genotype and a 26% increase compared to the CT genotype. *DGAT1* polymorphisms are closely linked to variations in lipid metabolism and oxidative stress markers [28,29]. The *DGAT1* gene encodes the enzyme diacylglycerol O-acyltransferase 1, which plays a crucial role in triglyceride synthesis by catalyzing the final step in the conversion of diacylglycerol (DAG) to triglycerides [30,31]. Variations in this gene, particularly the TT genotype, have been associated with altered lipid profiles, including increased triglyceride levels, which can contribute to the generation of reactive oxygen species ROS and subsequent oxidative stress [28]. Oxidative stress markers such as MDA, a byproduct of lipid peroxidation [32], are elevated in certain *DGAT1* genotypes, indicating higher levels of lipid oxidation. The TT genotype, for example, has been shown to have the highest MDA levels, suggesting a greater susceptibility to lipid peroxidation and oxidative damage. This increase in oxidative stress can trigger the compensatory upregulation of antioxidant enzymes such as GluRed and GPx, which are also observed to be elevated in the TT genotype. These findings suggest that *DGAT1* polymorphisms not only influence lipid metabolism by affecting triglyceride synthesis and storage, but also modulate the oxidative stress response in cells, highlighting the interconnectedness of lipid metabolism and oxidative stress in the context of this gene.

A bioactive compound analysis revealed that the taurine, carnosine, and anserine concentrations were highest in the TT genotype. Taurine levels were elevated by 38% compared to both the CC and CT genotypes, while the carnosine and anserine levels were 8% and 26% higher, respectively, in the TT genotype compared to the CC genotype. These bioactive compounds are known for their roles in reducing oxidative stress and enhancing muscle function [33], which may contribute to the adaptive responses observed in TT animals under oxidative stress conditions [9]. The lack of variation in coenzyme Q10 levels across *DGAT1* genotypes aligns with earlier research, suggesting that Q10 biosynthesis and regulation are relatively unaffected by *DGAT1* polymorphisms.

The observed variations in oxidative stress markers and bioactive proteins across different *DGAT1* genotypes can be linked to the enzymatic role of *DGAT1* in lipid metabolism and its impact on oxidative stress and inflammation. *DGAT1* catalyzes the final step in triglyceride synthesis, and its activity influences the accumulation of triglycerides, which in turn affects the cellular response to fatty acids (FAs). Notably, *DGAT1* has been shown to prefer monounsaturated fatty acids, such as oleoyl-CoA (18:1), over saturated fatty acids like palmitoyl-CoA [34]. This substrate preference suggests that *DGAT1* helps to protect cells from the toxic effects of excess FAs by incorporating them into triglycerides, thereby reducing the availability of free FAs that could lead to oxidative stress [35,36]. The protective role of *DGAT1* against inflammation is supported by evidence showing that transgenic MCK-*Dgat1* mice, when fed a high-fat diet, exhibit a reduced phosphorylation of JNK1 (p-JNK), a key component of inflammatory activation in skeletal muscle [37]. Similar protective effects have been observed with liver-specific overexpression of DGAT2, which induced hepatic steatosis without activating inflammatory markers such as p-JNK and p-NFkB, as seen in high-fat diet-induced steatosis [38]. Conversely, the chronic suppression of DGAT2 expression using a DGAT2-specific antisense oligonucleotide (ASO) has been associated with increased levels of ALT, a marker of hepatic inflammation, in mice with diet-induced hepatic steatosis [39]. These findings underscore the dual role of DGAT enzymes in managing both lipid metabolism and inflammation.

*LEP* Gene

Superoxide dismutase is a crucial antioxidant enzyme, categorized under oxidoreductases, which rely on metal cofactors at their catalytic core to neutralize oxidative stress [40,41]. SOD enzymes are widespread across various cellular compartments and play a pivotal role as the first line of defence against oxidative stress by catalyzing the conversion of superoxide anions (O_2_•−) into molecular oxygen (O_2_) and hydrogen peroxide (H_2_O_2_), the latter of which requires further detoxification [42]. The different types of SOD enzymes are distinguished by the metal ion cofactor in their active site, detailed as follows: Fe-SODs are generally located in the chloroplasts of plants and prokaryotes, Mn-SODs are found in the mitochondria and peroxisomes of both eukaryotes and prokaryotes, and Cu/Zn-SODs are predominantly found in the cytosol and extracellular spaces of eukaryotic cells [43]. In the context of the *LEP* gene, the observed elevation in malondialdehyde (MDA) levels in the CC genotype suggests a link between this genotype and increased oxidative stress [44]. The increase in oxidative markers, particularly MDA levels, is counterbalanced by heightened GPx activity in the CC genotype, indicating a robust antioxidant response in these animals. Furthermore, SOD activity was significantly higher in the CT genotype, exhibiting a 24% increase over the CC genotype and a 14% increase compared to the TT genotype. This finding suggests a genotype-dependent regulation of SOD that may operate independently of lipid peroxidation levels, as indicated by the MDA data. This underscores the complexity of antioxidant responses and the potential for distinct genetic influences on oxidative stress management among different genotypes [45]. The highest total antioxidant status (TAS) in the TT genotype further supports the idea of a genotype-specific antioxidant capacity, with the TT genotype demonstrating a 24% increase over the CC genotype and a 9% increase over the CT genotype, indicating a possible correlation between TAS and genotype-specific antioxidant mechanisms. This distinct regulation of SOD and overall antioxidant capacity across genotypes may be attributed to the differential localization and activity of SOD isoforms, with Cu/Zn-SOD (SOD1) in the cytosol, Mn-SOD (SOD2) in the mitochondria, and Cu/Zn-SOD (SOD3) in extracellular tissues, each contributing uniquely to the oxidative stress response in a genotype-dependent manner [46]. Understanding these variations could shed light on the underlying genetic mechanisms governing oxidative stress and antioxidant defences in livestock.

Bioactive compound levels associated with the *LEP* gene indicate that taurine levels remained stable across CC and CT genotypes but were slightly lower in the TT genotype. Carnosine levels were highest in the CC genotype, showing a 6% increase compared to the TT genotype, while anserine concentrations were highest in the TT genotype, with a 24% increase over the CC genotype and a 27% increase over the CT genotype. This suggests that the TT genotype may favour the accumulation of specific bioactive compounds such as anserine, which may play a role in modulating the oxidative stress response [47].

*SCD1* Gene

The *SCD1* gene plays a critical role in lipid metabolism, particularly in the biosynthesis of monounsaturated fatty acids (MUFAs) from saturated fatty acids [48,49,50]. The observed variations in oxidative stress markers and antioxidant enzyme activities among different *SCD1* genotypes highlight the gene’s influence on oxidative stress regulation and antioxidant defence mechanisms. The VA genotype’s association with the highest MDA levels suggests that this genotype may be more susceptible to lipid peroxidation compared to the AA and VV genotypes. MDA is a well-known marker of oxidative stress, particularly reflecting the extent of lipid peroxidation, which indicates cellular damage due to reactive oxygen species. The significant decrease in MDA levels observed in the VV genotype, compared to both the AA and VA genotypes, implies that the VV genotype might provide a protective effect against lipid peroxidation. This protection could be attributed to differences in the *SCD1* enzyme activity or the expression levels of other genes involved in lipid metabolism and ROS detoxification [51].

The AA genotype, which demonstrated the highest activities of GluRed, GPx, and SOD, suggests a robust and potentially more effective antioxidant defence system in this genotype. The elevated GluRed activity in the AA genotype, 18% higher than in the VA genotype and 50% higher than in the VV genotype, indicates a greater capacity for maintaining reduced glutathione (GSH) levels, a critical component of cellular antioxidant defences. Similarly, the highest GPx activity in the AA genotype suggests an enhanced ability to reduce hydrogen peroxide (H_2_O_2_) to water, thus preventing the accumulation of this potentially harmful ROS.

The SOD activity, which was 35% higher in the AA genotype compared to the VA genotype and 38% higher than in the VV genotype, further supports the notion of a more robust antioxidative response in the AA genotype. SOD is the first line of defence against superoxide radicals, catalyzing their conversion into hydrogen peroxide, which is subsequently reduced by GPx [52]. Therefore, the combined high activities of SOD, GPx, and GluRed in the AA genotype suggest a coordinated and efficient system for managing oxidative stress.

Total antioxidant status was also highest in the AA genotype, with a 9% increase over the VA genotype and a substantial 59% increase compared to the VV genotype. TAS reflects the cumulative action of all antioxidants present in the plasma and body fluids, indicating the overall capacity to neutralize free radicals [9]. The higher TAS in the AA genotype suggests that this genotype may have an enhanced overall ability to counteract oxidative stress, potentially due to the combined effects of increased antioxidant enzyme activities and possibly higher levels of non-enzymatic antioxidants.

These findings underscore the importance of the *SCD1* gene in modulating oxidative stress and antioxidant defences. The differential expression and activity of antioxidant enzymes across the *SCD1* genotypes could be a reflection of the gene’s influence on cellular redox homeostasis. The AA genotype, with its superior antioxidant enzyme activities and higher TAS, may confer a greater resilience against oxidative stress, potentially offering a protective advantage in conditions where oxidative stress is a significant factor. Conversely, the VA genotype’s higher MDA levels suggest a vulnerability to lipid peroxidation, which could have implications for the susceptibility of individuals with this genotype to oxidative stress-related conditions. Understanding these genotype-specific differences in antioxidant defences could inform breeding strategies aimed at enhancing oxidative stress resistance in livestock.

Regarding bioactive compounds, the AA genotype had the lowest taurine levels, with a 19% decrease compared to the CC and CT genotypes, and a 19% decrease compared to the VA and VV genotypes. Carnosine levels were highest in the AA genotype, showing a 4% increase compared to the VA genotype and a 10% increase compared to the VV genotype. Anserine levels were highest in the VV genotype, with a 13% increase compared to the AA and VA genotypes, indicating that the VV genotype may have a distinct metabolic profile favouring the accumulation of anserine.

## 5. Conclusions

The analysis of *DGAT1*, *LEP*, and *SCD1* gene polymorphisms reveals significant insights into how these genetic variations influence lipid metabolism, oxidative stress, and antioxidant enzyme activity.

*DGAT1* Gene: The TT genotype of *DGAT1* is associated with significantly higher levels of MDA, indicating increased lipid peroxidation and oxidative damage. This suggests that individuals with the TT genotype might be more susceptible to oxidative stress. This susceptibility is likely counteracted by an upregulation of antioxidant enzymes such as GluRed and GPx. The elevated levels of taurine, carnosine, and anserine in the TT genotype indicate adaptive mechanisms that help mitigate oxidative stress, suggesting a protective role for these bioactive compounds under oxidative conditions.

*LEP* Gene: For the *LEP* gene, the CC genotype is associated with the highest levels of MDA, indicating increased oxidative stress. Despite this, the higher activity of GPx and SOD in the CC genotype suggests a robust antioxidant response. Interestingly, SOD activity was highest in the CT genotype, while TAS was highest in the TT genotype. These variations imply that different *LEP* genotypes may regulate antioxidant responses differently, independent of MDA levels, potentially due to differences in the localization and activity of SOD isoforms.

*SCD1* Gene: The AA genotype of *SCD1* exhibits the highest activities of GluRed, GPx, and SOD, suggesting a more effective antioxidant defence system. The elevated activities of these enzymes in the AA genotype indicate a stronger capacity for managing oxidative stress. In contrast, the VA genotype shows the highest MDA levels, indicating a greater susceptibility to lipid peroxidation, while the VV genotype appears to offer some protection against oxidative damage, as reflected by lower MDA levels.

This study underscores the importance of researching oxidative stress in livestock, which occurs from an imbalance between reactive oxygen species and antioxidant defences. Identifying genetic variations affecting antioxidant enzyme activity, such as glutathione peroxidase and superoxide dismutase, is vital for breeding programmes aimed at improving oxidative stress resistance and enhancing livestock resilience.

## Figures and Tables

**Table 1:** Correlation coefficients between the biomarkers of oxidative stress and *DGAT1*, *LEP*, and *SCD1* in Hereford bulls.

	*DGAT1*	*LEP*	*SCD1*
MDA	0.075	−0.049	−0.024
GluRed	0.369 **	0.115	−0.318 **
GPX	0.056	−0.118	0.110
SOD	−0.038	0.090	−0.368 **
TAS	0.309 *	0.140	−0.275 *

*LEP*—leptin; *DGAT1*—diacylglycerol O-acyltransferase; *SCD1*—Stearoyl-CoA desaturase; MDA—malondialdehyde; GluRed—glutathione reductase; GPx—glutathione peroxidase; SOD—superoxide dismutase; TAS—total antioxidant status; ** Correlation significant at a level of 0.01 (two-sided). * Correlation significant at a level of 0.05 (two-sided).

**Table 2:** Correlation coefficients between bioactive proteins and genetic variants of *DGAT1*, *LEP*, and *SCD1* in Hereford bulls.

	*DGAT1*	*LEP*	*SCD1*
Taurine	−0.055	−0.578 **	−0.024
Coenzyme Q10	−0.078	−0.411 *	−0.037
Carnosine	−0.051	−0.278 *	−0.025
Anserine	−0.062	−0.514 **	−0.014

*LEP*—leptin, *DGAT1*—diacylglycerol O-acyltransferase; *SCD1*—Stearoyl-CoA desaturase ** Correlation significant at a level of 0.01 (two-sided). * Correlation significant at a level of 0.05 (two-sided).

## Data Availability

All data generated or analyzed during the study are included in this published article. The datasets used and/or analyzed in the current study are available from the corresponding author on reasonable request.

## Supplementary Materials

The following supporting information can be downloaded at: https://www.mdpi.com/article/10.3390/biom14101309/s1, Table S1: Characteristics of the bulls participating in the experiment. Table S2: Ingredients and chemical composition of transition and finishing rations fed in the finishing stage.
