# Supplementary material for: Genotype-Dependent Variations in Oxidative Stress Markers and Bioactive Proteins in Hereford Bulls: Associations with DGAT1, LEP, and SCD1 Genes
Source: Biomolecules. 2024 Oct 16;14(10):1309. doi: 10.3390/biom14101309 (PMC11506831; doi:10.3390/biom14101309)
Supplement: Supplementary file 1 [file biomolecules-14-01309-s001.zip › biomolecules-3232912-supplementary.pdf]

Table S1. Characteristics of the bulls participating in the experiment

		DGAT1			LEP			SCD1		
		CC (n=26)	CT (n=15)	TT (n=24)	CC (n=16)	CT (n=31)	TT (n=18)	AA (n=30)	VA (n=14)	VV (n=21)
**Meat characteristics [%]**										
**Collagen**	LSM	1.11^{A}	0.89^{A,B}	1.26^{B}	1.09	1.12	1.08	1.14^{a}	1.10^{b}	0.93^{a,b}
	SEM	0.025	0.017	0.032	0.046	0.032	0.060	0.028	0.033	0.209
**Fat**	LSM	3.95^{A,B}	4.25^{A,C}	5.41^{B,C}	4.35^{a,b}	3.99^{a,c}	4.00^{b,c}	3.89^{A,B}	4.35^{A,C}	3.08^{B,C}
	SEM	0.186	0.370	0.204	0.158	0.113	0.175	0.239	0.270	0.409
**Moisture**	LSM	70.99	69.88	70.22	70.65	70.97	70.82	71.14	70.53	71.83
	SEM	0.162	0.227	0.613	0.128	0.197	0.163	0.179	0.236	0.142
**Protein**	LSM	23.21	23.72	22.87	23.06	23.31	23.12	25.18^{A,B}	23.19^{A}	23.67^{B}
	SEM	0.073	0.307	0.185	0.167	0.088	0.121	0.113	0.092	0.186
**Salt**	LSM	0.96^{A,b}	0.76^{A,C}	0.81^{b,C}	0.94	0.92	0.97	1.07^{A,B}	0.87^{A,C}	0.64^{B,C}
	SEM	0.064	0.150	0.213	0.059	0.071	0.097	0.103	0.069	0.178
**Carcass weight [kg]**	LSM	365.15^{A,B}	338.83^{A,C}	370.26^{B,C}	358.78^{A,B}	367.92^{A,C}	361.92^{B,C}	372.30^{A,B}	358.92^{A,C}	365.28^{B,C}
	SEM	2.387	2.916	3.214	2.454	2.035	2.747	2.251	2.655	3.074

LSM-last square means; SEM-standard error; LEP-leptin, DGAT1-diacylglycerol O-acyltransferase; SCD1-Stearoyl-CoA desaturase; values with the same letters in the row differ significantly: upper case at $p \leq 0.01$; small case at $p \leq 0.05$.

Table S2. Ingredients and chemical composition of transition and finishing rations fed in the finishing stage.

Item	Ration	
**Ingredient composition, % as-fed**	1	2
**Grass silage**	77.9	55.8
**Rapeseed meal**	3.3	8.0
**Distillers grains**	9.5	21.2
**Grain mix (triticale and barley (50:50)**	8.6	14.3
**Minerals**	0.7	0.7
**Chemical composition:**		
**DM, %**	58.1	75.1
**CP, %**	13.5	16.6
**Fat, %**	3.0	4.7
**NEm, Mcal/kg DM**	1.6	2.2
**NEg, Mcal/kg DM**	1.1	1.5

Rations 1 = step-up diets fed for 7 d; ration 2 = finishing diet.
